# Developing and implementing a model of care for athletes living with disabilities: A protocol

**DOI:** 10.4102/sajp.v79i1.1868

**Published:** 2023-07-19

**Authors:** Siyabonga H. Kunene

**Affiliations:** 1 Department of Physiotherapy, Faculty of Health Sciences, University of the Witwatersrand, Johannesburg, South Africa; 2 Wits Sports and Health, Faculty of Health Sciences, University of the Witwatersrand, Johannesburg, South Africa

**Keywords:** model of care, healthcare services, rehabilitation, para-sports, South Africa

## Abstract

**Background:**

Athletes living with disabilities (ALWDs) face various challenges including stigma, discrimination, poor access to quality services and lack of funding. Their needs are also numerous. Despite the transformative promise of ‘Leave No One Behind’ of the United Nations’ 2030 agenda, people living with disabilities are still left behind.

**Objectives:**

To investigate the challenges and needs of ALWDs and to develop and implement a model of care (MoC), using South Africa as an example.

**Method:**

Our study will use a mixed-method design. The conceptual framework to manage this project is guided by a ‘process redesign framework’. Phase 1 will analyse the problems and needs of ALWDs. Phase 2 will include a review study to map the range of care strategies for athletes. Phase 3 will include a Delphi study to develop a suitable MoC. Phase 4 will involve the implementation of the MoC. Participants for phase 1 will include ALWDs. Phases 3 and 4 will include disability sports experts.

**Results:**

Our study will present the challenges and needs of ALWDs and propose a MoC.

**Conclusion:**

There is a need to think beyond disability and have robust discussions to challenge the way services are provided for ALWDs. Everyone has a role to play to help bring about changes that will improve the quality of life of these athletes.

**Clinical implications:**

A new MoC will assist in improving the quality of service for ALWDs.

## Background

### Disability at glance

There are over 1.3 billion (16%) people living with a disability (PLWD) worldwide (World Health Organization [WHO] 2021). About 80% of these come from low- and middle-income countries. Statistics of PLWDs are dramatically increasing and this is because of a rise in chronic health conditions, and demographic trends among many other causes (WHO 2021). According to Statistics South Africa (2011), about 7.5% of people in South Africa have disabilities. The global human movement, with its slogan #WeThe15%, has tried to ensure that PLWDs become visible and noticed in society. The United Nations 2030 agenda for sustainable development has also tried to promote PLWDs to a position where they are noticeable and not left behind (United Nations 2015). These initiatives are aimed at transforming the lives of PLWDs who represent 16% of the world’s population (WHO 2021).

Despite the transformative promise of ‘Leave No One Behind’ on the United Nations’ 2030 agenda, full inclusion of PLWDs remains a challenge in many countries including South Africa (Trani et al. 2020). Athletes living with disabilities (ALWDs) are still left behind (Rademeyer 2017). People living with disabilities are still facing challenges of stigma and discrimination among many other challenges (Trani et al. 2020). Trani et al. (2020) report that PLWDs in parts of South Africa face problems of poverty, poor nutrition and poor inclusion in labour markers. They also found that social stigma about disability was associated with various mental health issues including depression, low self-esteem, depressive mood, somatic indicators and negative feelings. People living with disabilities also have issues with the availability, accessibility, affordability and adequacy of services, for example problems with accessing healthcare, rehabilitation and education services (Trani et al., 2020). These are some of the issues that make full inclusion (in society e.g. service delivery) of PLWD a challenge.

### Sports and disability

Sports has the power to change the lives of PLWDs (United Nations 2011). It has the power to inspire them to do great things. Sports is a ‘double-edged sword’, which comes with many benefits including physical, behavioural, social, cognitive and psychological benefits (Malm, Jakobsson & Isaksson 2019). Sports participation of ALWDs in the Paralympics has increased over the years, from 16 competitors in 1948 (Stoke Mandeville Games) to 4328 in 2016 (Rio Paralympics) (Ghosh & Bhowmick 2018) and to 4403 in 2021 (Tokyo Paralympics) (Derman et al. 2023). There has been an increase in the number of sporting events that PLWDs participate in. Paralympians participated in about 22 sports and 23 disciplines in the 2020 Tokyo Paralympics (Derman et al. 2023).

The involvement of ALWDs has a long history. Sir Ludwig Guttman was one of the most significant figures in the history of disability sports (Ghosh & Bhowmick 2018). He believed that ‘by restoring activity in mind and body – by installing self-respect, self-discipline, a competitive spirit, and comradeship – sport develops mental attitudes that are essential for social reintegration’ (Brukner & Khan 2017). From the early times of Ludwig Guttmann, more and more sporting codes were then included in the games and Parasports gained popularity. Athletics, swimming, table tennis and basketball were a few sporting codes that gained popularity at the early start of Parasports. In 1976, people with vision impairments and limb deficiencies were then included. In 1980, the sporting code with athletes with cerebral palsy was formed (Legg & Steadward 2011).

The term ‘Olympics for disability’ was first used and then later the term ‘Paralympics’ was adopted. In 1989, the International Paralympic Committee (IPC) was then formed. Today, different disability groups are organised by various national and international organisations. The IPC is an international representative body for elite sports for ALDWs especially those with physical and visual impairments. Hearing-impaired athletes participate in Deaflympics which is organised by the international organisation called Comite International Sports de Sourds (CISS). International Sports Federation for Persons with Intellectual Disability (INAD-FID) is affiliated with IPC and represents elite athletes with intellectual disabilities (Legg & Steadward 2011).

Although participation in sports for PLWDs has been increasing over the years, these people still participate less in sports compared to able-bodied people (Ives et al. 2021; Jaarsma et al. 2013; McLoughlin et al. 2017). There is limited research conducted on the history and impact of sports on PLWD in South Africa (Rademeyer 2017). Rademeyer indicated that, during the apartheid era, disability sports had to endure abuse and social problems. This unfortunate situation contributed to poor sports participation among PLWD, especially among the black population.

Since 1994, disability sport in South Africa has grown faster than some codes in able-bodied sports (Rademeyer 2017). Rademeyer indicated that some of the ALWDs have become internationally renowned sporting icons; they have put disability sports in the limelight. According to Rademeyer (2017), South Africa is starting to recognise and care better for ALWDs. Athletes living with disabilities are being treated almost in the same way as able-bodied athletes. In my experience with working and travelling with ALWDs, I have also noted the improvements made by the South African government when it comes to organising and managing Para games. However, there is still more work to be conducted to improve the lives of these athletes. Athletes living with disabilities in South Africa are still facing numerous challenges as compared to able-bodied athletes (Rademeyer 2017). Issues of stigma, discrimination and access to services are some of the challenges that still need to be addressed (Rademeyer 2017; Trani et al. 2020). Despite these challenges, ALWDs are showing resilience. Jim Abbott, a former one-handed baseball pitcher once said:

It’s not the disability that defines you, it’s how you deal with the challenges the disability presents you with. We have an obligation to the abilities we do have, not the disability. (Brukner & Khan 2012)

### Barriers and facilitators to sports participation

There are various drawbacks to sports participation among ALWDs. People living with disability experience various barriers to sports participation including lack of understanding and awareness of how to include people with a disability in sports; limited opportunities and programmes for participation, training, and competition; lack of accessible facilities; limited accessible transportation and limited access to information and resources (Ives et al. 2021). Ives et al. (2021) further showed that these barriers are personal, physical, environmental, social, economic and political in nature.

Disability and general health are some personal barriers to sports participation among many people, leading them to a lack of self-confidence and motivation to participate in sports (Ives et al. 2021; McLoughlin et al. 2017). The severity and type of disability and health problems are associated with participation in sports. People with spinal cord injuries for example have an increased risk of developing cardiovascular issues which may affect their ability to participate (Ives et al. 2021; Shields & Synnot 2016). These are the personal barriers that PLWDs face.

The environment can be another barrier to sports participation among PLWDs. One of the most prominent issues that affects participation among PLWDs is the lack of accessibility to sports facilities and services, especially for people in middle- and low-income countries (Ives et al. 2021; Jaarsma et al. 2013). People living with disabilities who stay far from sports facilities or services may find it difficult to get to and from the facility and are therefore more likely to not participate (Ives et al. 2021). In addition to that, the available facilities may not be physically accessible for wheelchair users including issues like narrow openings of doorways, lack of ramps and the like (McLoughlin et al. 2017).

Studies have shown that some PLWDs are not willing to participate in sports because of social stigma and negative attitudes in society. A study conducted for children with disabilities showed that some children are bullied when participating in sports and physical activity. This is more than enough to discourage PLWDs from participating in sports (Ahmed et al. 2018; Jaarsma et al. 2013; Shields & Synnot 2016).

Adaptive equipment used for sports such as specialised wheelchairs and prosthetic limbs can be expensive and some people with disabilities may not be able to afford them (Erdmann 2018). According to Erdmann (2018), the cost of transportation to and from training and sporting facilities has also proved to be a significant barrier to participation in sports for people with physical disabilities. Therefore, funding can be a serious barrier to sports participation among ALWDs, especially athletes in poor communities (Huus et al. 2021). Lack of funding can then lead athletes to use inappropriate equipment which can lead to injuries and poor performance (Huus et al. 2021).

Lack of skilled training personnel and coaches has also impacted sports participation for people with physical disabilities (Ahmed et al. 2018; Ives et al. 2021; McLoughlin et al. 2017).

However, there are also facilitators of sports participation for people with physical disabilities. As much as it is important to understand the barriers to sports participation, it may be just as important or even more important to understand strategies that have already proven successful in encouraging participation in sports for people with disabilities. A passion and desire for sports and motivation are significant facilitators (Craig et al. 2019; Jaarsma et al. 2013). Some have been influenced into participating in sports because of the health benefits (Jaarsma et al. 2013; McLoughlin et al. 2017).

Support from family and friends can be a powerful psychological motivator for participation. Family members may also provide support in terms of transporting an athlete to and from sports facilities (Jaarsma et al. 2013). A good relationship between a coach and an athlete can be quite a powerful motivator for participation and can reduce dropouts (Oladunni, Lyoka & Goon 2015).

Participation in sports for people with physical disabilities allows them to interact with others and form relationships outside of their family. This is important and therefore influences people to participate (Craig et al. 2019; Oladunni et al. 2015; Shields & Synnot 2016).

Proximity to sporting facilities and accessibility encourages participation among ALWDs (Jaarsma et al. 2013; McLoughlin et al. 2017; Oladunni et al. 2015). Athletes from poor-resourced homes who live far from sports facilities may find it challenging to access these facilities. To increase participation among ALWD, sports facilities need to be made accessible.

### A need for change

Disability sport has been characterised by a series of challenges as discussed in previous paragraphs. There is a need for sustainable interventions to address stigma, discrimination, barriers to sports participation and issues of poor access to services. In an in-depth semi-structured interview study conducted in South Africa, Swartz et al. (2018) indicate that there is still work to be done to further explore the challenges faced by ALWDs, particularly in South Africa. Swartz et al. (2018) showed a need for rehabilitation professionals to find better ways to care for these athletes. A comprehensive intervention is needed to address the medical, rehabilitation, human performance and sociocultural issues facing ALWDs. There is a need to further understand and examine opportunities for sports participation (Swartz et al. 2018). Addressing the challenges that ALWDs face is critical. If injuries and risks are not addressed, athletes end up training and competing with their injuries, thus compromising their quality of life (QoL) (Kunene, Taukobong & Ramklass 2021). If barriers to sports participation among PLWDs are not addressed, many people with disabilities will continue to be disadvantaged, thus losing the benefits that come with sports participation.

The limitation of rehabilitation services is another problem that discourages participation and usually results in athletes withdrawing from participation in sports (Kunene et al. 2021). South Africa is one of the countries that still has a low level of sports participation among PLWDs (Rademeyer 2017). Rehabilitation services are also not readily available and accessible to most communities. There is a need to make services available, accessible, affordable, adequate and appropriate (Conchar et al. 2016; Kunene et al. 2021). There is a need to integrate diversity, equity and inclusion as key pillars for change. To break this vicious circle of challenges ALWDs are facing, something must be done to ensure there is substantial psycho-social support and anti-stigma policies anchored in local cultural values. (Conchar et al. 2016; Rademeyer 2017). Athletes living with disabilities and the community will need to be engaged effectively for an intervention to be effective.

There is currently no suitable and comprehensive model of care (MoC) that exists in the current literature (as far as the author is concerned) to better address the barriers and needs of ALWDs. A need exists in the country for a MoC to improve health services delivered to ALWDs. There is currently a need to provide best-practice care and services for these athletes as they progress through the stages of their conditions, injuries or events. Athletes living with disabilities in South Africa deserve to get the proper care, on time, by the appropriate team, and in the right place. Our study proposes the development and implementation of a suitable and comprehensive MoC to bring about change and transformation in the way healthcare services are delivered for ALWDs in South Africa. The objectives include:

- To gain insight into barriers and facilitators of sports participation among ALWDs.
- To describe the needs of ALWDs.
- To determine the prevalence and risks of sports-related injuries among ALWDs.
- To map the range of current care strategies used for ALWDs.
- To identify criteria and items to be considered for inclusion in the MoC.
- To develop a suitable MoC in collaboration with experts.
- To implement the developed MoC and explore the experiences of model adopters and beneficiaries.

## Conceptual framework

**FIGURE 1:**
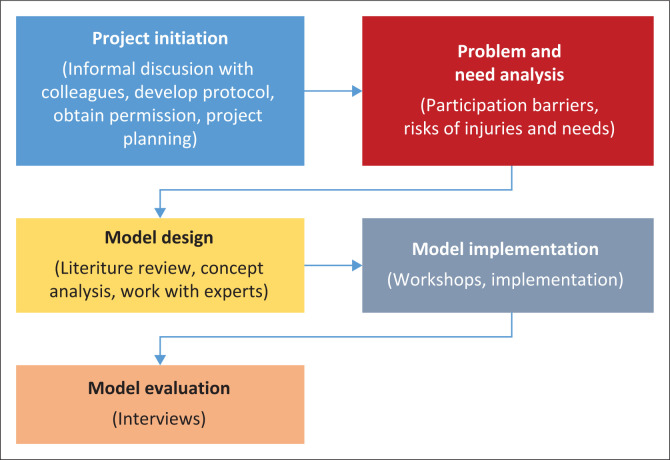
Conceptual framework for the development and implementation of a model of care.

Figure 1 shows a conceptual framework illustrating the proposed process of development and implementing a MoC for our study. This conceptual framework will help the first author and all the stakeholders to design and support the implementation of the proposed innovative MoC for ALWD. According to Davidson et al. (2006), a MoC is a ‘thorough and comprehensive plan for providing health care services based on evidence and established standards’. In the context of this project, a MoC is a plan for providing healthcare services for ALWDs. According to Davidson et al. (2006), there are crucial stages in the MoC development. These are planning, development, implementation, evaluation and sustainability. Our study will be guided by these crucial stages and will be conducted in four phases as outlined in Figure 1. Phase 1 is project initiation that has already taken place. This phase included an informal discussion with colleagues, development of a protocol, obtaining permission to conduct our study and project planning. Our study is currently at phase 2, a problem and needs analysis phase. This phase is about gaining insight into barriers and facilitators of sports participation, a description of the needs of athletes and determining the prevalence and risks of injuries. Phase 3 is about the actual development of the MoC. The process will include conducting a literature review (to establish current intervention strategies used in sports), concept analysis and obtaining consensus among experts. Phase 4 will involve the implementation of the model. At the end of the implementation phase, the model will then be evaluated by exploring the experiences of model adopters and beneficiaries.

## Methods

Our study will use a mixed-method (qualitative and quantitative) design. It will consist of a mix of cross-sectional exportation, semi-structured interviews, scoping review, Delphi and focused ethnography. The following sub-sections discuss the methods for each sub-study included in our study.

### Establishing barriers and facilitators

This will be a cross-sectional exploratory study. The population will include South African Paralympic multi-coded athletes affiliated with the South African Sports Association for people living with Physical Disability (SASAPD). Only athletes with physical disabilities will be included. These will include athletes with spinal cord injuries (congenital or acquired), limb deficiency or amputation, cerebral palsy and Les Autres (such as muscular dystrophies and syndromic conditions). Male and female athletes who are ≤ 18 years of age will be included and who can also read and understand English. Athletes with cognitive disabilities will be excluded.

According to the SASAPD database, about 1892 athletes are registered. A suitable representative sample size required will be 320 (with a confidence level of 95%, margin of error of 5% and response distribution) according to the online sample size calculator by Raosoft (http://www.raosoft.com/samplesize.html).

A questionnaire developed by Jaarsma et al. (2013) will be modified and used to collect the data. Their study looked at barriers and facilitators of sports in the Dutch Paralympics. The content and face validity of this tool will be evaluated by obtaining the input of at least five experts (medical professionals) who are well versed in working with ALWDs. These will include sports physiotherapists, sports physicians, sports psychologists and biokineticists. After the questionnaire has been scrutinised by the experts and revised by the first author, it will then be piloted for suitability among at least 10% of the same population. The questionnaire will be in English. A reading level of 8th and 9th school grade English (score of 70 – 60) will be used. This refers to plain English, which is easily understood by a 13–15-year-old learner. According to the information received from SASAPD, most registered athletes can read and understand English.

To recruit participants, the first author will send an email invitation with study information and an online questionnaire link to SASAPD and request them to disseminate our study information and questionnaire link to athletes who meet the inclusion criteria. Participants will then contact and respond directly to the first author. The RedCap programme will be used as the online platform to administer this questionnaire. Please see the attached information that will be sent to SASAPD.

Descriptive (frequencies, mean, median, interquartile ranges) and inferential statistics (analysis of variance [ANOVA], Chi-square, regression analysis) will be obtained from the data collected.

### Determining the needs

A semi-structured interview will be conducted to determine the needs of participants. Interviews will be conducted via the online platform (Zoom). Participants for this sub-study will be the same participants for the sub-study mentioned earlier. The number of participants to be recruited will depend on data saturation. To recruit participants, the first author will send an email invitation to SASAPD with study information and request them to disseminate our study information to athletes who meet the inclusion criteria. Participants will then contact and respond directly. The first author will then schedule an online interview meeting with the consenting participants. Consent to participate will be obtained from each participant before the data-collection process commences. Consent to record the interview will also be obtained. Each interview session is scheduled to last for approximately 20 min. The names of the participants will be coded numerically in the transcripts. The data will be analysed thematically using an inductive process. The process of analysing data will be as per the following steps: (1) familiarisation with the data by reading and re-reading the original transcript, while listening to the audio recording, (2) development of themes and subthemes from the concepts and categories from data obtained and (3) defining and naming the themes and subthemes. A few rounds of discussions may need to take place between reviewers, where they will compare their findings to improve the credibility of the process.

### Determining injury prevalence and risks

Self-developed online surveys will be used to determine (1) the injury prevalence among all sporting codes and (2) potential risk factors involved in sports injury causation among the common sporting codes (athletics, swimming, cycling). Modifiable and non-modifiable extrinsic factors will be determined. Modifiable factors will include sets of questions under the following themes (1) rules and regulations, coaching education and/or training, playing time, playing surface and equipment. Non-modifiable factors will include sets of questions under the following themes (1) type of sports, (2) sport context, (3) weather conditions, (4) level of play, (5) time of the season and (6) playing position. Three questionnaires will be developed for athletes participating in athletics, swimming and cycling. Each sporting code will be given a specific questionnaire. The tools will be reviewed by a panel of experts for content and face validity. A pilot study will also be conducted to validate the tools before they can be used. The questionnaires will be in English. Participants who can read and understand English will be included. A reading level of 8th and 9th school grade English (score of 70 – 60) will be used. This refers to plain English, which is easily understood by a 13–15-year-old learner. To recruit participants, the first author will send an email invitation to SASAPD with study information and request them to disseminate our study information to athletes who meet the inclusion criteria. Participants will then contact and respond directly to the first author. The RedCap programme will be used as the online platform to administer this questionnaire. Participants will be visited in their training venues for physical screening. Descriptive (frequencies, mean, median, interquartile ranges) and inferential statistics (ANOVA, chi-square, regression analysis) will be obtained from the data collected.

### Current care strategies

The purpose of this sub-study is to map the range of current care strategies for ALWDs. This will be a scoping review conducted using Joanna Briggs Institute (JBI) guidelines. Before the study commences, the first author will register the title of this sub study on the Open Science Framework. This will ensure that there are no title duplications. The PRISMA GROUP guidelines and the JBI reviewer’s manual will be used to structure the methodology of this review according to the guidelines for a scoping review. The PRISMAScR checklist will be used to ensure quality. Joanna Briggs Institute SUMARI software will also be used to systematically conduct each step of the scoping review process.

The Population, Concept, Context (PCC) framework will be used to structure the inclusion criteria.

- Types of participants: Studies involving ALWDs will be included in this study. Participants may be of any age, gender or ethnicity and may participate in any level of sports.
- Concept: This proposed review will consider the inclusion of studies that looked at injury prevention and rehabilitation and other intervention strategies for ALWDs. This may include rehabilitation programmes or protocols, strategies and models.
- Context: This proposed scoping review will consider inclusion of any context in which injury prevention and rehabilitation and other intervention strategies for ALWDs are recommended.

Our review will consider the inclusion of all studies published within the past 20 years that are available in full text. All studies published in peer-reviewed journals and grey literature will be considered for inclusion because of the relatively limited quantity of studies on this topic.

The literature search strategy will involve a three-step process. Step one will involve the reviewers conducting a preliminary search of MEDLINE (PubMed) and CINAHL using the following keywords: injury*, prevention*, rehabilitation, strategy*, care, solution*, model*, athlete*, sports and disability*. The titles, abstracts and keywords of relevant articles will then be screened by the first author to identify keywords to be used in the MeSH terms for an in-depth search. Step two will involve a more expansive search using the established MeSH terms following step one of the search strategy. The databases of CINAHL, MEDLINE (via PubMed), Cochrane Database of Systematic Reviews and PEDro, EBSCOhost, CINAHL, SPORTDiscus, PubMed, COCHRANE and Scopus will be searched in step two. Step three will involve the screening of reference lists of extracted articles to acquire additional articles that may be of relevance. Duplicate articles will be identified and removed as needed.

Two reviewers will be involved in the search and reviewing of the literature. The first reviewer will select studies based on their title and abstract that are deemed worthy of critical appraisal. A full-text review will then be conducted by two independent reviewers (first author and research assistant) of included articles. Reviewers will exclude articles that are deemed to no longer meet the inclusion criteria based on an in-depth review. The discrepancy between reviewers regarding which articles will be included will be resolved through discussion and based on consensus. If an agreement is not reached by the two independent reviewers, the judgment of a third reviewer will be deemed final. The first author as well as the second reviewer will be included in the data-extraction process to ensure there is no risk of bias.

Both reviewers will conduct data extraction independently in congruence with the JBI guidelines for scoping reviews. The following information will be extracted: author(s), date of publication, study design and level of evidence, aim(s) of study, population size and description, the screening tool(s) used to predict injury, the accuracy of screening tool(s) if investigated, the definition of injury, diagnosis of injury and findings. A pilot data-extraction process will be conducted on at least three articles by at least two reviewers to test the appropriateness of the data-extraction form.

Results will be presented through a narrative summary of the extracted data. Where appropriate, this narrative synthesis will be represented in images and tables to assist in data presentation.

### Model development

A Delphi method will be used to develop the MoC for ALWDs in South Africa. A panel of experienced sports clinicians, sports management and sports technicians involved in disability sports will be headhunted and recruited by independent healthcare professionals to participate in this study. These experts will include two sports physicians, two sports physiotherapists, two biokinetics, two podiatrists, two dieticians, two psychologists, two sports managers/coaches, two sports technicians, two representatives from disability associations, athlete representatives and two community leaders. The aforementioned professionals must have at least 10 years of clinical experience in working with ALWDs. Emails will be used as a form of communication between the first author and the participants. All participants will be provided with the relevant study information, an invitation to participate and a consent form. Participants’ demographic data will be collected with the aid of a questionnaire that will be developed by the first author. The data that will be collected will include gender, age, profession, current position and years of professional experience.

The Delphi process will include three rounds:

- Round 1: The first author will use evidence from relevant articles and data from the preliminary sub-studies to generate criteria and items to be included in the MoC. The identified items will be converted into a questionnaire. The questionnaire will be disseminated to Delphi participants for review, grading and comments. Delphi participants will be asked to review and rate each item using a five-point Likert scale, ranging from 0 to 4 (4 = strongly agree, 3 = agree, 2 = disagree, 1 = strongly disagree, and 0 = neutral). A space for comments will be provided in the questionnaire.
- Round 2: In this round, the first author will analyse the responses from round 1. Scores of each participant will be reviewed and items that do not reach the pre-determined conceptual score of at least three out of four will be excluded. In this round, the list of items will be reviewed taking into consideration the suggestions from the experts. A revised list of items and round 1 scores will be shared with the same panel via email. Instructions from round 1 will be given again for this round.
- Round 3: The first author will analyse round 2 responses and exclude the items that will score less than three out of four. A final list of items will then be prepared and sent back to Delphi participants for the final rating. This will be the last round if no more statement items are needed to be removed. This will mean that a consensus is reached.

The first author will then draft the MoC and prepare a report. Descriptive statistics will be used to present the demographic profile of the Delphi participants and their responses to the questionnaire. The acceptable level of consensus will be set at > 80%. Previous Delphi studies accepted a similar level of consensus (Diamond et al. 2014; Henderson & Rubin 2012; Slade et al. 2014; Vogel et al. 2019).

### Implementation

After the model has been developed and consensus among experts has been obtained, the first author will then implement the model and explore the experiences of model adopters (SASAPD, SASCOC, sports medical practitioners, sports officials) and beneficiaries (ALWDs).

To implement the newly developed MoC, workshops will be conducted for relevant stakeholders for example SASAPD, SASCOC, sports clinicians, sports officials and ALWDs. The purpose of the workshops will be to educate participants about the developed model. After 3 months of conducting workshops, the model will then be piloted in Gauteng province for a period of 6 months.

After 6 months, the experiences of model adopters and beneficiaries will be explored to establish the impact of the model. A focused ethnography method will be used. Ethnography is about ‘making cultural inferences from people’s communications, actions, and artefacts’ (Spradley 2016). The culture of interest in our study will include the sports community (ALWDs, sports clinicians, sports leadership) in diverse sports community healthcare sites across Gauteng. A focus group methodology will be used. To ensure diversity in the selection of participants, a purposive sampling method will be used. Representation from urban and rural settings will be considered. An interview schedule will be developed and used to guide the interview process. The schedule will comprise a set of questions that will be exploring participants’ experiences during the model implementation, challenges faced, perceptions of the model in practice and criteria for defining the successful adoption of the model. Feedback from five experts (experienced researchers in the field) will be obtained regarding the developed interview schedule. This will be done to validate the interview schedule.

The first author will visit and email various sports healthcare sites, SASAPD and SASCOC to recruit participants. An appointment (date, time and site) will be made with each participant. On the day of data collection, participants will be provided with a study information sheet, focus group guidelines and written consent form. The first author will facilitate and audio record (with participants’ consent) the interviews. After each interview, data will be saved on a password-protected computer. Data will only be accessed by the first author and the data analyst. The names of the participants will be coded numerically in the transcripts. The data will be analysed thematically, using an inductive process. The process of analysing data will be as per the following steps: (1) familiarisation with the data by reading and re-reading the original transcript, while listening to the audio recording, (2) development of themes and subthemes from the concepts and categories from data obtained and (3) defining and naming the themes and subthemes. A few rounds of discussions may need to take place between reviewers, where they will compare their findings to improve the credibility of the process.

### Ethical considerations

This study has obtained ethical clearance from the Human Research Ethics Committee (Medical) of the University of the Witwatersrand (ethical clearance number: M220120). Permission to conduct the study was also obtained from the South African Sports Association for the Physically Disabled. Study information will be given to prospective participants and written consent to participate in our study will be solicited.

## Discussion

The 2030 Agenda for sustainable development which was adopted by 93 member states of the United Nations and the general assembly in September 2015, is a new road map that emphasises leaving no one behind (United Nations 2019). It is a roadmap to address some of the most urgent priorities for countries in the United Nations. According to the United Nations, these include ensuring well-being for all, reducing inequality in all its dimensions and promoting inclusion for all, ensuring availability and affordable services for all. This transformative agenda ensures that PLWDs are also not left behind. This means that people with the fewest development opportunities should be reached first (United Nations 2019). Sustainable development is possible when everyone is included.

In trying to align with the United Nations’ 2030 agenda, the South African government developed the national development plan to address issues facing PLWDs (National Planning Commission 2012). The national development plan proposes to address many issues including the issue of lack of healthcare services. It aims to ensure accessible and affordable access to quality health care for all while promoting health and well-being. It also aims to ensure that PLWDs are not excluded when it comes to the provision of services. It emphasises that this group be prioritised. In South Africa, disability sports has not yet fully received the recognition it deserves (Rademeyer 2017) and healthcare services have not been easily accessible and affordable for PLWDs (National Planning Commission 2012; Rademeyer 2017).

The aim of our study is aligned with the South African national development plan. It is to develop a ‘custom-made’ intervention (a MoC) to deal with some of the barriers to sports participation for ALWDs, particularly the issues of poor access and affordability of healthcare and rehabilitation services. An MoC will propose the best practice and services for ALWDs as they progress through various stages of their sports-related conditions (injuries or events). It will ensure that these athletes get appropriate care timeously and by the right team (Davidson et al. 2006).

The methodology chosen to embark on our study will ensure that the goals are realised. Because of the complex nature of our study, a mixed methods design was chosen as the most suitable design to develop and implement a MoC (Davidson et al. 2006). As described in the methods section, in our study, the authors will combine various elements of qualitative and quantitative research approaches to determine the barriers to sports participation, the needs of ALWDs, mapping the range of existing caring strategies used in sports and developing and implementing the actual MoC.

The process of developing and implementing the MoC has been informed by the practical guidelines published by the Agency for Clinical Innovation (2013) and the work conducted by Davidson et al. (2006). The guiding principles include the following processes: project initiation, diagnosing a problem, designing a solution, implementation and sustainability (Agency for Clinical Innovation 2013). To implement our proposed MoC will be a complex process. It will involve changing the way healthcare services are delivered for ALWDs. Therefore, the ‘process redesign framework’ will need to be used to assist with conceptualising, mapping refining and continuing to improve processes of healthcare (Smith et al. 2014).

As much as there is no existing MoC for ALWDs, it is worth noting that we will not be developing this model from scratch. We will build on what other scholars have done in the past. There are various models, frameworks and theories that have been developed with the aim of improving health service delivery in general. One of the objectives of our study is to review the existing literature and map the current strategies used to care for ALWDs. Our study will become the foundation on which a new MoC will be established.

Various models of disability exist. The psychological model of disability (Johnston 1997) is one of them. This model deals only with the psychological and emotional effects of disability. Affirmative or affirmation model (Swain & French 2000) is another model. This deals with non-tragic views about disability and impairment which deals with positive identities for PLWDs. Smart and Smart in 2006 described an environmental model which deals with the environment of PLWDs. They also described a functional model that focuses on functional performance and limitations of PLWDs. Samaha (2007) also introduced personal traits of PLWDs within a social model. Kuppers (2009) proposed a rhizomatic model that relates to a variety of experiences. Bolt (2015) on the other hand suggested a happiness-related model. Levitt (2017) developed a model of disability that focuses on actions of PLWDs. His model is called ‘active’. All these models deal more with social identities and effects of actions of PLWDs. There are not specific and comprehensive models that deals with challenges and needs of ALWDs. Our study seeks to close this existing gap.

Medical and rehabilitation models for athletes in general have been changing with an increased focus on individualised and context-based care. The traditional medical model is being discouraged because it has the medical doctor as the primary contact healthcare practitioner with subsequent referral to other healthcare professionals (physiotherapists, psychologists, dieticians, etc.) (Brukner & Khan 2017). This model lacks the aspect of the consideration of different points of view of all professionals involved. It does not promote integration of ideas from all professionals. In my experience, this model is unfortunately still used in various clinical settings in South Africa including sports medicine settings. The early sports and exercise medicine model seems better compared to the medical model (Brukner & Khan 2017), but it needs to be improved as well in order to recognise its multidisciplinary nature. The improved version of this model still emphasises the reductionist approach where each discipline is still operating in its own isolated specialist field, participially in South Africa. The sports and exercise medicine model does not promote the holistic management of athletes. It also lacks effective communication integration and understanding when it comes to arriving at decisions. Dijkstra et al. (2014) proposed a new Integrated Performance Health Management and Coaching Model which appears to be more comprehensive. In this recent model, decisions are made as a team, not in isolation, taking into consideration the best evidence and performance preferences.

There is still a need to improve the way health services are delivered among athletes, especially for disadvantaged and marginalised athletes (Ives et al. 2021; Swartz et al. 2018). The models discussed above have tried to cover as much territory as possible, but they have not incorporated community-based, transdisciplinary and task-shifting approaches to accommodate various contexts. The new Integrated Performance Health Management and Coaching Model seems ideal, but it has only been illustrated by describing the organisational and implementation strategies used by the United Kingdom athletics (Dijkstra et al. 2014). Our study is aimed at developing a MoC for ALWDs that is comprehensive enough to recognise other contexts. South Africa and other African countries present with a unique sports history and management (Conchar et al. 2016). Therefore, ALWDs in these countries need custom-made ways of delivering health services.

## References

[CIT0001] Agency for Clinical Innovation, 2013, *Understanding the process to develop a model of care – An ACI framework*, viewed n.d., from https://aci.health.nsw.gov.au/__data/assets/pdf_file/0009/181935/HS13-034_Framework-DevelopMoC_D7.pdf.

[CIT0002] Ahmed, B.S., Lamy, M., Cameron, D., Artero, L., Ramdial, S., Leineweber, M. et al., 2018, ‘Factors impacting participation in sports for children with limb absence: A qualitative study’, *Disability and Rehabilitation* 40(12), 1393–1400. 10.1080/09638288.2017.1297496 28286964

[CIT0003] Bolt, D., 2015, ‘Not forgetting happiness: The tripartite model of disability and its application in literary criticism’, *Disability & Society* 30(7), 1103–1117. 10.1080/09687599.2015.1071240

[CIT0004] Brukner, P. & Khan, K., 2012, *Brukner & Khan’s clinical sports medicine: Volume 1 Injuries*, 4th edn., p. 960, N.S.W. McGraw-Hill Education Australia, North Ryde.

[CIT0005] Brukner, P. & Khan, K., 2017, *Brukner & Khan. Clinical sports medicine: Volume 1 injuries*, 5th edn., pp. 769–804, N.S.W. McGraw-Hill Education Australia, North Ryde.

[CIT0006] Conchar, L., Bantjes, J., Swartz, L. & Derman, W., 2016, ‘Barriers and facilitators to participation in physical activity: The experiences of a group of South African adolescents with cerebral palsy’, *Journal of Health Psychology* 21(2), 152–163. 10.1177/1359105314523305 24607923

[CIT0007] Craig, P.J., Barcelona, B., Aytur, S., Amato, J. & Young, S.J., 2019, ‘Using inclusive sport for social change in Malawi, Africa’, *Therapeutic Recreation Journal* 53(3), 244–263. 10.18666/trj-2019-v53-i3-9720

[CIT0008] Davidson, P.M., Halcomb, E., Hickman, L., Philips, J.L. & Graham, B., 2006, ‘Beyond the rhetoric: What do we mean by a “model of care”?’, *Australian Journal of Advanced Nursing* 23(3), 47–55, viewed n.d., from https://www.researchgate.net/publication/7210210_Beyond_the_rhetoric_What_do_we_mean_by_a_’Model_of_Care’/citations#fullTextFileContent. 16568879

[CIT0009] Derman, W., Runciman, P., Eken, M., Boer, P.H., Blauwet, C., Bogdos, M. et al., 2023, ‘Incidence and burden of injury at the Tokyo 2020 Paralympic Games held during the COVID-19 pandemic: A prospective cohort study of 66045 athlete days’, *British Journal of Sports Medicine* 57, 63–70. 10.1136/bjsports-2022-106234 36588428

[CIT0010] Diamond, I.R., Grant, R.C., Feldman, B.M., Pencharz, P.B., Ling, S.C., Moore, A.M. et al., 2014, ‘Defining consensus: A systematic review recommends methodologic criteria for reporting of Delphi studies’, *Journal of Clinical Epidemiology* 67(4), 401–409. 10.1016/j.jclinepi.2013.12.002 24581294

[CIT0011] Dijkstra, H.P., Pollock, N., Chakraverty, R. & Alonso, J.M., 2014, ‘Managing the health of the elite athlete: A new integrated performance health management and coaching model’, *British Journal of Sports Medicine* 48(7), 523–531. 10.1136/bjsports-2013-093222 24620040 PMC3963533

[CIT0012] Erdmann, W.S., 2018, ‘Equipment and facilities adapted for disabled people in recreation and sport’, *MedCrave Online Journal of Applied Bionics and Biomechanics* 2(1), 9–13. 10.15406/mojabb.2018.02.00038

[CIT0013] Ghosh, S.S. & Bhowmick, S., 2018, ‘A review study on Paralympic games’, *International Journal of Sports and Physical Education* 4(1), 19–24. 10.20431/2454-6380.0401005

[CIT0014] Henderson, E.J. & Rubin, G.P., 2012, ‘Development of a community-based model for respiratory care services’, *BMC Health Services Research* 12, 193. 10.1186/1472-6963-12-193 22776670 PMC3474150

[CIT0015] Huus, K., Schlebusch, L., Ramaahlo, M., Samuels, A., Berglund, I.G. & Dada, S., 2021, ‘Barriers and facilitators to participation for children and adolescents with disabilities in lowland middle-income countries – A scoping review’, *African Journal of Disability* 10, 1–10. 10.4102/AJOD.V10I0.771 PMC8008013 33824860

[CIT0016] Ives, B., Clayton, B., Brittain, I. & Mackintosh, C., 2021, ‘“I’ll always find a perfectly justified reason for not doing it”: Challenges for disability sport and physical activity in the United Kingdom’, *Sport in Society* 24(4), 588–606. 10.1080/17430437.2019.1703683

[CIT0017] Jaarsma, E. A., Geertzen, J.H.B., de Jong, R., Dijkstra P. U. & Dekker R., 2013, ‘Barriers and facilitators of sports in Dutch Paralympic athletes: An explorative study, *Medicine and Science in Sports* 24(5), 830–836. 10.1111/sms.12071 23662691

[CIT0018] Johnston, M., 1997, ‘Integrating models of disability: A reply to Shakespeare and Watson’, *Disability & Society* 12(2), 307–310. 10.1080/09687599727407

[CIT0019] Kunene, S., Taukobong, N. & Ramklass, S., 2021, ‘Community-based rehabilitation implementation framework to address patellofemoral pain amongst runners in under-resourced communities: Delphi consensus’, *South African Journal of Physiotherapy* 77(1), a1531. 10.4102/sajp.v77i1.1531 PMC8252162 34230899

[CIT0020] Kuppers, P., 2009, ‘Toward a Rhizomatic model of disability: Poetry, performance, and touch’, *Journal of Literary & Cultural Disability Studies* 3(3), 221–240. 10.1353/jlc.0.0022

[CIT0021] Legg, D. & Steadward, R., 2011, ‘The Paralympic Games and 60 years of change (1948–2008): Unification and restructuring from a disability and medical model to sport-based competition’, *Sport in Society* 14(9), 1099–1115. 10.1080/17430437.2011.614767

[CIT0022] Levitt, J.M., 2017, ‘Developing a model of disability that focuses on the actions of disabled people’, *Disability & Society* 32(5), 735–747. 10.1080/09687599.2017.1324764

[CIT0023] Malm, C., Jakobsson, J. & Isaksson, A., 2019, ‘Physical activity and sports – Real health benefits: A review with insight into the public health of Sweden’, *Sports* 7(5), 127. 10.3390/sports7050127 31126126 PMC6572041

[CIT0024] McLoughlin, G., Fecske, C.W., Castaneda, Y., Gwin, C. & Graber, K., 2017, ‘Sport participation for elite athletes with physical disabilities: Motivations, barriers, and facilitators’, *Adapted Physical Activity Quarterly* 34(4), 421–441. 10.1123/apaq.2016-0127 28985104

[CIT0025] National Planning Commission, 2012, *National development plan – Our future – Making it work*, The Presidency, Republic of South Africa, viewed n.d., from https://www.gov.za/sites/default/files/gcis_document/201409/ndp-2030-our-future-make-it-workr.pdf.

[CIT0026] Oladunni, B., Lyoka, P.A. & Goon, D.T., 2015, ‘Perceived motivational factors influencing students with disabilities towards sports participation in Amathole district, Eastern Cape Province, South Africa’, *African Journal for Physical, Health Education, Recreation & Dance* 21(4:2), 1389–1401, viewed n.d., from https://hdl.handle.net/10520/EJC182292.

[CIT0027] Rademeyer, C., 2017, ‘Sport for people with disabilities as factor in reshaping the post-apartheid South African sporting society’, *Journal for Contemporary History* 42(1), 81–98. 10.18820/24150509/JCH42.v1.5

[CIT0028] Rademeyer, C., 2017, ‘Sports for people with disabilities as factor in reshaping the post-aparthied south africaan sporting society’, *Journal Contemporary History* 40(1), 81–98. 10.18820/24150509/JCH42

[CIT0029] Samaha, A.M., 2007, ‘What good is the social model of disability?’, *University of Chicago Law Review* 74(4), 1251–1308. 10.2307/20141862

[CIT0030] Shields, N. & Synnot, A., 2016, ‘Perceived barriers and facilitators to participation in physical activity for children with disability: A qualitative study’, *Boston Medical Center Pediatrics* 16(1), 989–997. 10.1186/s12887-016-0544-7 PMC4717582 26786677

[CIT0031] Slade, S.C., Dionne, C.E., Underwood, M. & Buchbinder, R., 2014, ‘Standardised method for reporting exercise programmes: Protocol for a modified Delphi study’, *British Medical Open* 4(12), e006682. 10.1136/bmjopen-2014-006682 PMC4281530 25550297

[CIT0032] Smart, J.F. & Smart, D.W., 2006, ‘Models of disability: Implications for the counseling profession’, *Journal of Counseling and Development* 84(1), 29–40. 10.1002/j.1556-6678.2006.tb00377.x

[CIT0033] Smith, L.M., Ashok, M., Morss Dy, S., Wines, R.C. & Teixeira-Poit, S., 2014, *Contextual frameworks for research on the implementation of complex system interventions*, Agency for Healthcare Research and Quality (US), Rockville, Maryland, US. 24783308

[CIT0034] Spradley, J.P., 2016, *The ethnographic interview*, Waveland Press, Long Grove, Illinois, United States.

[CIT0035] Statistics South Africa, 2011, Census 2011: ‘Profile of persons with disability in South Africa’, viewed n.d., from https://www.statssa.gov.za/publications/Report-03-01-59/Report-03-01-592011.pdf.

[CIT0036] Swain, J. & French, S., 2000, ‘Towards an affirmation model of disability’, *Disability & Society* 15(4), 569–582. 10.1080/09687590050058189

[CIT0037] Swartz, L., Bantjes, J., Knight, B., Wilmot, G. & Derman, W., 2018, ‘They don’t understand that we also exist: South African participants in competitive disability sport and the politics of identity’, *Disability and Rehabilitation* 40(1), 35–41. 10.1080/09638288.2016.1242171 27756168

[CIT0038] Trani, J., Moodley, J., Anand, P., Graham, L. & Thu Maw, M.T., 2020, ‘Stigma of persons with disabilities in South Africa: Uncovering pathways from discrimination to depression and low self-esteem’, *Social Science* & *Medicine Journal* 265, 113449. 10.1016/j.socscimed.2020.113449 PMC7576188 33183862

[CIT0039] United Nations, 2011, *Panel discussion on sports for inclusive development: Sports, disability and development: Key to empowerment of persons with disabilities and their communities*, UN Headquarters, New York, NY, viewed n.d., from https://www.un.org/development/desa/disabilities/panel-discussion-on-sports-for-inclusive-development-sports-disability-and-development-key-to-empowerment-of-persons-with-disabilities-and-their-communities-27-june-2011-1-15-to-2-30-p-m-confer.html.

[CIT0040] Vogel, C., Zwolinsky, S., Griffiths, C., Hobbs, M., Henderson, E. & Wilkins, E., 2019, ‘A Delphi study to build consensus on the definition and use of big data in obesity research’, *International Journal of Obesity* 43(12), 2573–2586. 10.1038/s41366-018-0313-9 30655580 PMC6892733

[CIT0041] World Health Organization, 2021, *Disability*, viewed n.d., from https://www.who.int/news-room/fact-sheets/detail/disability-and-health.

